# Evolution of *Aspergillus oryzae* before and after domestication inferred by large-scale comparative genomic analysis

**DOI:** 10.1093/dnares/dsz024

**Published:** 2019-11-22

**Authors:** Naoki Watarai, Nozomi Yamamoto, Kazunori Sawada, Takuji Yamada

**Affiliations:** 1 Department of Life Science and Technology, Tokyo Institute of Technology, Tokyo 152-8550, Japan; 2 Gurunavi, Inc., Tokyo 100-0006, Japan

**Keywords:** *Aspergillus oryzae*, *Aspergillus flavus*, comparative genomics, domestication

## Abstract

*Aspergillus oryzae* is an industrially useful species, of which various strains have been identified; however, their genetic relationships remain unclear. *A. oryzae* was previously thought to be asexual and unable to undergo crossbreeding. However, recent studies revealed the sexual reproduction of *Aspergillus flavus*, a species closely related to *A. oryzae*. To investigate potential sexual reproduction in *A. oryzae* and evolutionary history among *A. oryzae* and *A. flavus* strains, we assembled 82 draft genomes of *A. oryzae* strains used practically. The phylogenetic tree of concatenated genes confirmed that *A. oryzae* was monophyletic and nested in one of the clades of *A. flavus* but formed several clades with different genomic structures. Our results suggest that *A. oryzae* strains have undergone multiple inter-genomic recombination events between *A. oryzae* ancestors, although sexual recombination among domesticated species did not appear to have occurred during the domestication process, at least in the past few decades. Through inter- and intra-cladal comparative analysis, we found that evolutionary pressure induced by the domestication of *A. oryzae* appears to selectively cause non-synonymous and gap mutations in genes involved in fermentation characteristics, as well as intra-genomic rearrangements, with the conservation of industrially useful catalytic enzyme-encoding genes.

## 1. Introduction

*Aspergillus oryzae* is an industrially important species mainly used in the manufacture of fermented foods in East Asia because of its strong amylase and protease activities.^1^ Particularly, in Japan, *tane-koji* (seed rice malt) manufacturers use various strains that are sold to companies producing fermented foods. These strains show diversity in colour and fermentation function and are managed for different applications such as in *sake*, *miso* (soybean paste), and *shoyu* (soy sauce). However, the relationship between the diversity of *A. oryzae* species and genetic factors remains unclear.

In 2005, the whole genome of *A. oryzae* RIB40, a wild-type strain, was sequenced.^2^ Comparative genomic analysis of the whole genomes of *Aspergillus nidulans* and *Aspergillus fumigatus* revealed that the *A. oryzae* genome was 7–9 Mb larger.^2^^,^^3^ However, genes in newly acquired regions are only minimally expressed under normal conditions,^4^ and most of their functions remain unknown, particularly for genes not directly involved in fermentation.

*Aspergillus flavus* and *A. oryzae* are genetically very closely related, with their genomes showing 99.5% similarity in coding regions,^5^ and numerous comparative analyses have been performed between these species. *Aspergillus flavus* is an important species linked to food safety, as some strains produce fungal toxins, particularly aflatoxin,^6^^,^^7^ and it has historically been distinguished from *A. oryzae* based on morphological differences and toxicity.^7^^,^^8^ In addition, some *A. oryzae* strains contain all or parts of the aflatoxin biosynthetic gene cluster, although they are non-aflatoxigenic.^6^

Some researchers suggested that *A. oryzae* can be detoxified and differentiated from *A. flavus* by domestication.^1^^,^^9^^,^^10^ Based on the phylogenetic analysis of 11 genes,^9^ comparative analysis of the aflatoxin gene cluster,^11^^,^^12^ and single-nucleotide polymorphism (SNP) analysis of the whole genome,^13^*A. oryzae* was shown to form a monophyletic clade derived from one clade of *A. flavus*.

*Aspergillus oryzae* and *A. flavus* have long been considered asexual species with no sexual reproduction cycle.^14^ However, recent studies of *A. flavus* revealed that sexual reproduction occurs in laboratory and field environments.^15^^,^^16^ Genome analysis also showed that the two species contain a nearly complete gene set necessary for sexual reproduction.^3^^,^^17^ All strains of *A. oryzae* and *A. flavus* possess one mating type (MAT type) locus in the genome, at which either MAT1-1 or MAT1-2 is encoded.^17^^,^^18^ However, complete sexual reproduction has not been confirmed in *A. oryzae*. Breeding currently carried out by *tane-koji* manufacturers utilizes a single strain with mutations or recombination, but crossbreeding has not been successful. Genome analysis suggested that recombination occurred between the ancestors of *A. oryzae* based on the linkage disequilibrium between MAT types and the phylogeny of a single gene.^19^

In this study, to uncover genomic diversity and evolutionary relationships among *A. oryzae* isolates, we acquired 82 industrial strains from five independent Japanese *tane-koji* manufacturers in different locations and conducted whole-genome sequencing to determine their draft genomes. For the classification of these strains, we performed orthologue clustering of predicted genes from each genome, phylogenetic tree inference of the chromosomal genome, and chromosome recombination analysis. Through these analyses, we hypothesized that *A. oryzae* strains have undergone multiple inter-genomic recombination events between *A. oryzae* ancestors, and that evolutionary pressure by *A. oryzae* domestication is extremely limited to intra-genomic mutations and rearrangements. Moreover, we identified genes that are mutated/duplicated/deleted within clades, which might reflect the fact that Japanese *tane-koji* manufacturers have passaged their strains to prevent changes in industrially useful traits in parallel with breeding.

## 2. Materials and methods

A full description of the methods, including software versions and parameters, is available in Supplementary Data (‘supplementary_methods.pdf’).

### 2.1. Sample collection and DNA preparation

For genomic sequencing, 82 *A. oryzae* and three *Aspergillus sojae* (as an out group) industrially used strains were collected from five independent *tane-koji* manufacturers in Japan (Supplementary Table S1). *Tane-koji* manufacturers have their own isolates and have not shared them for several decades. Whole genomic DNA was extracted using ‘Extraction method5’.^20^ Yatalase was used for some samples (Supplementary Table S1).

### 2.2. Genome sequencing and assembly

For genome assembly, fragmented genome libraries were prepared based on 350 bp (for run no. 1) and 550 bp (for run no. 2–5) on average and sequenced on an Illumina HiSeq2500 system using 150 bp (for run no. 1) and 250 bp (for run no. 2–5) paired-end runs. Quality filtering and assembly of the paired-end reads were performed with Platanus.^21^ The scaffolds aligned to bacterial genomes or the mitochondrial genome of RIB40 were removed. The reference primer sequences for the MAT type^17^ were mapped to the genome sequences with bowtie2.^22^

### 2.3. Gene prediction and orthologous clustering

Next, 152 genomic scaffolds (85 from our samples and 67 from NCBI GenBank) of the newly sequenced or NCBI GenBank *Aspergillus* strains were used (Supplementary Table S2). Gene coding regions were predicted using two methods, namely, GeneMark-ES^23^ and AUGUSTUS^24^ for *ab initio* prediction, and GMAP^25^ for reference-based prediction, and these were combined with EVidenceModeler.^26^ The predicted protein sets were evaluated with BUSCO.^27^ Orthologue clustering was performed with OrthoFinder.^28^ Orthogroups (OGs) were annotated with GhostKOALA^29^ for protein function and InterProScan^30^ for protein motifs/domains.

### 2.4. Comparative genomics

Alignments of degapped gene sequences (DGSs) of single-copy OGs (SCGs), which were common to 152 protein sets, were generated with MAFFT^31^ and tandemly concatenated. Maximum likelihood-based phylogenetic inference was performed with RAxML.^32^ Similarly, a concatenated gene tree of 19 SCGs in the aflatoxin biosynthetic cluster was generated. To test the neutrality of the mutation, revised coding sequences were generated from the alignments by clade/species and synonymous, non-synonymous, and gap mutations were counted. Chromosomal duplications and deletions were inferred by direct read mapping to the RIB40 genome with bowtie2. Depths were calculated with samtools.^33^

## 3. Results and discussion

### 3.1. Genome sequencing and assembly

The number of scaffolds (>1,000 bp) and total lengths were 36–256 and 35.9–38.66 Mb for *A. oryzae* and 45–71 and 40.0–40.1 Mb for *A. sojae*, respectively (Supplementary Table S2). The two draft genome sizes were not significantly different from those reported previously.^2^^,^^34^ All samples were sequenced at depths of ≥100, but the depths of some samples from runs no. 3, no. 4, and no. 5 showed relatively low average coverage of the final scaffolds because the genomic DNA of *Actinobacteria* (*Corynebacterium*) used to produce yatalase was contaminated.

### 3.2. Gene prediction and evaluation

Gene prediction for our sample showed that the number of predicted genes of *A. oryzae* and *A. sojae* were 11, 196–11, 716 and 13, 309–13, 317, respectively (Supplementary Table S2, column S). To confirm the accuracy of assembly and gene prediction, the predicted gene set and reference gene set were analyzed with BUSCO. In the complete genome of RIB40 (GCF_000184455.2), the score of the gene set was 98.6% in our prediction pipeline.

### 3.3. Orthologue clustering

By orthologue clustering, 15,614 OGs were created from 1,882,788 proteins, while 1,395 proteins became unclustered. Of these, 3,951 were common SCGs. We succeeded in the annotation of the KEGG KO and protein domains/motifs at 28.6% (4,465/15,614) and 88.0% (13,752/15,614) (Supplementary Table S3), and assigned locus IDs starting with ‘AO’ as defined previously^2^ to 13,006 OGs clustered with the *A. oryzae* RIB40 reference proteins (s01-m08-r29 or s01-m09-r06).

### 3.4. Comparative genomics

#### 3.4.1. Phylogenetic tree with concatenated genes

**Figure 1:**
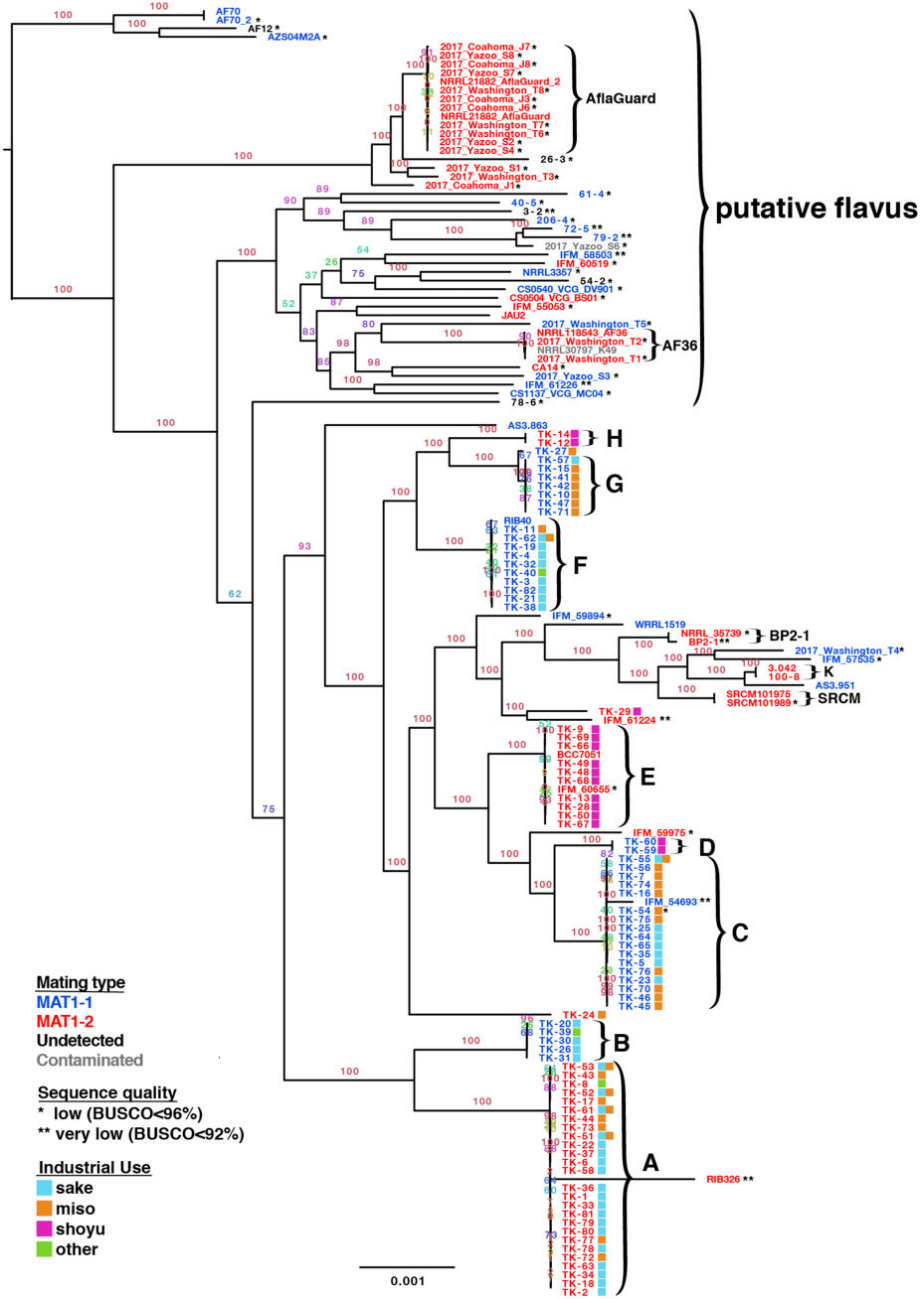
Phylogenetic tree inferred by concatenated DGSs, focusing on *Aspergillus oryzae* and *Aspergillus flavus*. A–H: clade names for Japanese industrial strains defined in this study. AflaGuard, AF36, BP2-1, K, and SRCM are temporary clade names.

The length of the sequence of the concatenated DGSs of 4,361 SCGs was 5,677,852 columns (bp), corresponding to approximately 15% of the total genome length. We confirmed that *A. oryzae* was monophyletic and nested in a clade of *A. flavus* (Fig. 1, original full figure: Supplementary Fig. S1). This is consistent with the results of previous studies.^9^^,^^11–13^ In contrast, some putative *A. flavus* strains (WRRL1519, NRRL35739, IFM54693, IFM57535, IFM59975, IFM60655, and 2017 Washington T4) were nested in the *A. oryzae* clade. However, WRRL1519,^35^ NRRL35739, and the IFM strains^36^ were confirmed as non-aflatoxigenic with *A. oryzae*-type aflatoxin gene clusters (also confirmed in this study). Thus, considering the location in the phylogenetic tree and toxigenicity, these strains were reclassified as *A. oryzae*.

Industrial strains of *A. oryzae* formed several clades, showing an intra-cladal DGS dissimilarity within 0.01%. For further analysis, we defined clades A–H to which Japanese industrial strains of *A. oryzae* belong. Although *koji* manufacturers do not share their strains, interestingly, many strains clustered in the same clade. Particularly, clade E included several strains provided by four different *tane-koji* manufacturers, one strain collected in Thailand (BCC7051),^37^ and one strain as a clinical isolate from Japan. In contrast, some Chinese and Korean industrial strains, namely, AS3.951, 100.8/3.042 (China),^38^ BP2-1, and the two SRCM strains (Korea), formed a group with a relatively large distance from the Japanese industrial strains. These strains were very closely related to each other but should be classified into different clades because of their different MAT types.

Industrial *A. oryzae* strains were not clustered based on their industrial uses. However, all strains in clades D, E, and H (and a single strain TK-29) were those used in *shoyu* production. Strains used for other purposes such as in *miso*, *sake*, *sake*/*miso*, *mirin*, or other products were mixed in the same clade, which is consistent with classification by appearance and enzymatic activity.

#### 3.4.2. MAT type

Of the 152 genomic sequences, MAT regions were uniquely detected as either MAT1-1 or MAT1-2 from 146 sequences but not from six downloaded sequences (Fig. 1). We examined the presence/absence of genes in each strain and identified OG0012491/AO090020000089 as MAT1-1 and OG0012281 + OG0012282 as the MAT1-2 gene (these two were contiguous, and thus possibly merged into one gene). The identified region of MAT1-1 was consistent with that reported previously.^19^ The genes unique to each MAT type were only these MAT genes. All strains included in the same clade showed the same MAT type. Linkage disequilibrium was observed between the topology of the phylogenetic tree of concatenated genes and MAT type (e.g. A&B and G&H). This strongly suggests that clade divergence was caused not by mutation but rather by the recombination of different strains.

#### 3.4.3. Putative genetic recombination

**Figure 2:**
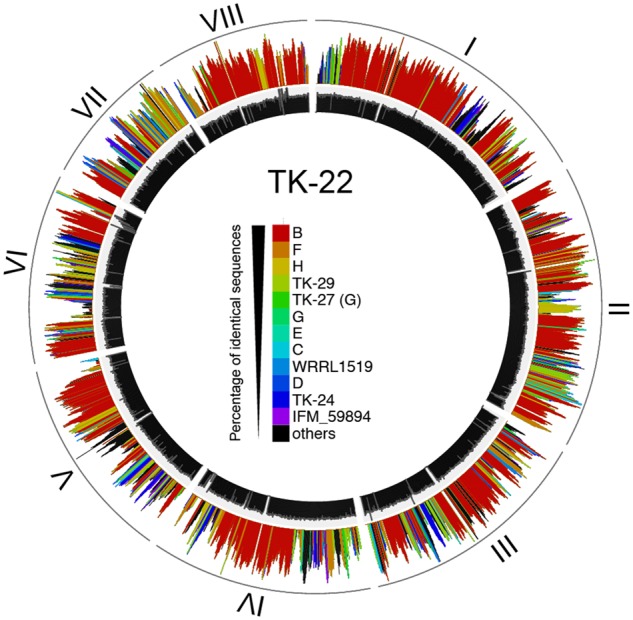
Visualized chromosomal mixture of TK-22 of clade A. I–VIII represent chromosome numbers. Sequence depths are shown in the inner circle with black bars. Chromosomal mixture is shown in the outer circle with coloured bars, where heights represent similarity scores (percentage of identical gene sequences) and colours represent the closest clade.

In addition to MAT type, as an example of linkage disequilibrium, we also found that the phylogenetic tree of the concatenated gene showed a different topology from those of individual genes. For example, on comparing the phylogenetic tree of *ytk6*/OG0002894/AO090023000584 to that of *mdlB*/OG0004275/AO090701000644, the combinations of clades with the same sequence were different (Supplementary Fig. S2-1A and B). In addition, the phylogenetic trees of adjacent genes showed a similar topology. For each strain, we identified the closest clade along the chromosomal positions (Supplementary Methods 2.4.3). As a result, the genomes of all clades of *A. oryzae* and *A. flavus* exhibited high mosaic structures; regardless of the clades, the closest clade differed depending on the chromosomal position. For example, TK-22 (as a representative strain of clade A) shared 46% of the exact same gene sequences with clade B, but the homologous genes were distributed in a mosaic manner (Fig. 2, unsmoothed figure: Supplementary Fig. S3-1). From the distribution of similarity scores, we can see that some non-syntenic regions against clade B could be syntenic regions of clade F (Supplementary Fig. S3-5). This means that clades closer on the concatenated gene tree had larger proportions of homologous regions, and the pattern of syntenic regions depended on the clade to be compared. We also detected locally exclusively homologous regions among distant clades (Supplementary Fig. S2-2). This suggests that clade divergence was caused by multiple recombination events in multiple strains, at least as many times as the number of clades, and not by passage mutation in only one ancestral strain.

In the *A. oryzae* clade, most genome regions were represented as a mixture with other *A. oryzae* clades; particularly, some were highly homologous to the *A. flavus* clade and vice versa. Thus, we cannot completely exclude the possibility of recombination between two species after speciation. However, because the exclusively homologous regions between the two species were very small, the strain used in this study might have been insufficient. Because our samples were biased toward practically used strains, information about wild-type strains of *A. oryzae* is lacking. Thus, the genomic mosaicism shown in this study does not directly represent the frequency of recombination. By analyzing more strains, the mosaic structure might be simplified and recombination processes in the clades could be clarified.

In addition, because our samples were human-managed strains, it might be possible to track whether hybridization occurred among them. However, none of the clades appeared to be expressed as a mixture of two other clades. In contrast, there were some *A. flavus* strains for which genomes were represented by a mixture of two or three other strains (Supplementary Fig. S3-2). Interestingly, the genome of *A. oryzae* TK-27, a strain maintained by a *tane-koji* manufacturer for more than six decades and the use of which started in the 2000s, had an unusual structure; 85% or more of the genome was homologous to clade G, to which the MAT types were also identical, while some regions were closer to those of the other clades (Supplementary Fig. S3-3). A Chinese strain, AS3.951, and the strains in clade K showed a similar pattern, although they had different MAT types (Supplementary Fig. S3-4). However, they were not a clear mixture of any strain pair. Based on these results, we consider that the domestication process influenced the evolution of *A. oryzae* mostly through rearrangements within a single genome, and rarely via sexual recombination.

### 3.5. Influence of domestication

#### 3.5.1. Evolutionary hypothesis

Our results highlighted the fact that the ancestor of *A. oryzae* underwent multiple complex recombination events. In contrast, considering that no simple recombination mixtures between strains from the *tane-koji* manufacturer were observed and many strains belonged to the same clade with wild-type RIB40, clinical isolates, or the strain from Thailand, humans might have chosen strains from nature as suitable for brewing and maintained them without crossbreeding. Therefore, the influence of domestication on the evolution of *A. oryzae* likely appeared only after clade divergence, suggesting that the domestication process does not contribute to genetic recombination.

**Figure 3:**
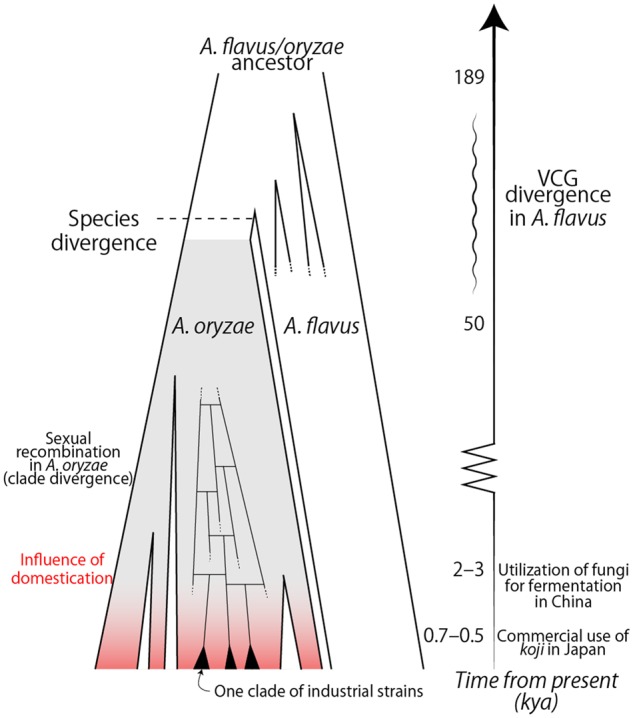
Hypothesis for the influence of domestication on the evolution of *Aspergillus oryzae.*

In previous reports, vegetative compatibility group (VCG) divergence in *A. flavus* was estimated to have occurred 50,000–189,000 years ago.^19^ VCG is a self-identification system, and there are at least 13 VCGs in *A. flavus*.^39^ Considering that *A. oryzae* is monophyletic and nested in one of the clades of *A. flavus* based on the phylogenetic tree, and that *A. oryzae* is one type of VCG, the speciation of *A. oryzae* and *A. flavus* might have occurred contemporaneously. Domestication and industrial utilization of *koji* (rice malt) began in China over 3,000–2,000 years ago, and stocking and selling of these products began in Japan 700–500 years ago.^1^ Therefore, domestication likely began influencing the evolution of *A. oryzae* in the last 3,000 or 700 years after clade divergence (Fig. 3).

In a previous study, mutational pressure was estimated by comparing the SNP frequencies of *A. flavus* and *A. oryzae*^13^ or by comparative genomics of RIB40 and RIB326.^40^ According to such studies, mutations tend to accumulate in non-synteny blocks (NSBs) and sub-telomeric regions. However, these estimates are considered to reflect the influence of selection pressure in nature during the period from species or clade divergence to domestication. Therefore, we focused on recently accumulated mutations or gene duplications/deletions by comparing them within each clade.

#### 3.5.2. Intra-cladal gene variants

We calculated the numbers of inter- and intra-cladal mutations in coding sequences for 14,711 SCGs present in 101 strains with BUSCO scores of 96 or more (Supplementary Method 2.4.5). There were a few intra-cladal mutations; in total, 265 synonymous mutations, 528 non-synonymous mutations, and 93 gap mutations were found in 201, 453, and 70 genes, respectively (Supplementary Table S4, 1–3), of which synonymous mutations were not prevalent enough for neutrality tests within each gene (Supplementary Table S4-4). Remarkably, however, the odds of the total number of intra-cladal non-synonymous/synonymous mutations were significantly higher than those of inter-cladal mutations in *A. oryzae* and much higher than those of intra-species mutations in *A. flavus*, with a similar tendency for gap/synonymous mutations (Table 1). This tendency was almost the same between synteny blocks (SB) and NSBs with *A. nidulans*/*A. fumigatus* (Supplementary Table S4-5).

**Table 1:** Total number of mutations in coding sequences of *Aspergillus oryzae* and *Aspergillus flavus*

			Synonymous mutations	Non-synonymous mutations	Gap mutations
*A. oryzae*	Intra-clade	Counts	265	528	93
		Odds		2.9*	20.8*
	Inter-clade	Counts	76,543	66,001	1,795
		Odds		1.3*	1.4*
*A. flavus*		Counts	79,068	53,922	1,336
		Odds		(1.0)	(1.0)

* *P* < 1.0E-10.

These results suggest that the influence of domestication, reflected by intra-cladal mutations and somewhat by inter-cladal mutations, causes the accumulation of non-neutral mutations changing gene functionality, such as loss of function. Even though there were no genes with sufficient number of inter-cladal mutations for the calculation of odds or *P* values (Supplementary Table S4-4), we found 1–4 intra-cladal non-synonymous or gap mutations among all the named genes (Table 2). For example, both intra-cladal non-synonymous and gap mutations were found in two annotated genes, specifically *wA*/OG0003175/AO090102000545 and *laeA*/OG0001970/AO0900030000489. *A. oryzae wA* is an industrially important polyketide synthase gene, an orthologue of *A. nidulans wA*, which is required for the synthesis of a green pigment.^41^ The down-regulation of the *A. oryzae wA* gene leads to the production of white conidia.^42^ Non-synonymous or gap mutations in *wA* were only found in some white mutants of clade A, C, and G used for *miso* production wherein the control of product color is important. *laeA* is a global regulator of secondary metabolism in *Aspergillus*,^43^ and it is also involved in the production of kojic acid.^44^

**Table 2:** Annotated genes with intra-cladal non-synonymous/gap mutations

RIB40_s01-m09-r06	Symbol	Annotated features
Genes with intra-cladal non-synonymous mutations		
AO090003001208	*amyR*	Regulatory protein that indirectly affects the production of hemicellulolytic and cellulolytic enzymes, likely through carbon catabolite repression-mediated control
AO090003000489	*laeA*	Methyltransferase; global transcriptional regulator of secondary metabolic gene clusters; required for kojic acid gene regulation and biosynthesis
AO090009000638	*steA*	Orthologue of *Ste12p* with a predicted role in the regulation of transcription
AO090001000237	*veA*	Orthologue of *Aspergillus nidulans* VeA, a global gene regulator involved in light-sensitive control of differentiation and secondary metabolism; positively regulates penicillin production in *Aspergillus oryzae*
AO090003000491	*rpbA*	Predicted RNA polymerase II largest subunit; has 25 repeats in its C-terminal domain
AO090026000360	*gprD*	Family A G-protein coupled receptor (GPCR)-like
AO090001000439	*schA*	Ser/Thr protein kinase related to the PKA catalytic subunit
AO090001000512	*cyaA*	Adenylate cyclase
AO090003001305	*aglB*	Putative alpha-galactosidase; expression altered by *manR* disruption but not by ManR overexpression
AO090003001507	*tglA*	Triacylglycerol lipase with a role in the degradation of triglycerides
AO090001000445	*CYP505A3*	Cytochrome P450 monooxygenase
AO090005000070	*CYP620H3*	Cytochrome P450 monooxygenase; involved in 7-hydroxycoumarin production
AO090012000465	*CYP620H9*	Cytochrome P450 monooxygenase; involved in 7-hydroxycoumarin production
AO090038000488	*csyC*	Putative type III polyketide synthase
AO090011000926	*dffA*	l-Ornithine N5-oxygenase; enzyme required for the biosynthesis of an iron-chelating compound, deferriferrichrysin; siderophore biosynthesis
AO090001000009	*wykN*	Non-ribosomal peptide synthase (NRPS) involved in the synthesis of a dipeptidyl peptidase IV 2 inhibitor
AO090102000632	*can1*	Has domain(s) with predicted role in amino acid transport, transmembrane transport, integral component of membranes, membrane localization
AO090103000127	*bglF*	Secretory aryl beta-glucosidase
AO090003001144	*vti1*	T-SNARE
AO090701000589	*chsB*	Chitin synthase; required for normal hyphal growth and conidiation
AO090026000337	*sec31*	Vesicle coat complex COPII, subunit; expression increased in MAT1-2 strain compared with that in MAT1-1 strain
AO090102000545	*wA*	Hydroquinone: oxygen oxidoreductase; orthologues have a role in asexual spore wall assembly, melanin biosynthesis, pathogenesis, and pigment metabolism
Genes with intra-cladal gap mutation		
AO090102000545	*wA*	(Above)
AO090003000489	*laeA*	(Above)
AO090009000612	*amdA*	Sequence-specific DNA-binding transcription factor

Interestingly, a few mutations in catabolic enzymes were considered industrially useful; moreover, no intra-cladal non-synonymous or gap mutations were found in known proteolytic enzyme-encoding genes. In contrast, there were several non-synonymous/gap mutations in genes involved in expression, signaling, secondary metabolites, secretion/transporters, and cell traits (Table 2). Therefore, the domestication process might have contributed to altering the traits of strains while maintaining the activity of industrially useful catalytic enzyme-encoding genes by applying selective pressure to those peripheral genes rather than enzyme-encoding genes. This might reflect the fact that Japanese *tane-koji* manufacturers have continued to passage their strains to prevent changes in their traits in parallel with breeding.

#### 3.5.3. Intra-cladal gene duplication

We estimated gene duplication/deletion by direct read mapping and calculation of the normalized depth, equal to the copy number of genes (Supplementary Table S5). As a result, we found 221 OGs (117 SCGs) with different estimated copy numbers in at least one clade and 179 OGs (20 SCGs) in more than two clades. We performed statistical analysis on KEGG BRITE annotation but found no significant feature (number of total detection > 1, *P* = 0.05, Fisher’s exact test), suggesting that selection pressure for gene duplication is not explained by gene function. The estimated copy numbers of OG0000041/PF03221/Tc5 transposase and OG0000321/PF14529/endonuclease-reverse transcriptase, which are transposon-derived genes, had changed more than twice as compared with that in RIB40; in particular, the clade B and G strains showed 15–20 times the estimated copy number. Furthermore, rRNA genes exhibited a wide range in estimated copy number (e.g. it varied 13–48-fold among eight strains of clade C without bacterial contamination from yatalase). However, the selective pressure on rRNA gene duplication is hard to estimate, because a previous report showed strain-dependent copy number variation in rRNA genes in *A. fumigatus* by quantitative PCR,^45^ suggesting that the duplication of rRNA genes is likely to occur in the natural environment. The estimated copy number of the tRNA gene also tended to vary, and intra-cladal change was observed at 102/276 tRNA gene loci.

As a general trend, changes in the sequence depth were more frequent in the sub-telomeric region (Supplementary Fig. S4-1, Chr. I, Fig. S4-2). Change in depth was also observed in the sub-centromeric regions, which represents changes in the number of non-coding repeats in the unassembled region. In contrast, in some strains, a 60–70-kbp region containing tRNA genes (e.g. Supplementary Fig. S4-1, Chr. III/V) was duplicated, which might have drastically altered transcription or translation.

Three copies of α-amylase (*amyA*/*amyB*/*amyC*/OG0011956) have been detected in RIB40.^2^ We found both intra-cladal and inter-cladal variation in the duplication number of OG0011956 (α-amylase) as follows: three to four copies in clade A, two to three copies in clade D/E, two copies in clade B, and four copies in clade G. Similarly, we found one copy in TK-24/TK-29 and four copies in TK-27. Moreover, the *A. sojae* strains TK-83, TK-84, and TK-85 had one copy, which is consistent with previous studies.^34^^,^^46^

#### 3.5.4. Aflatoxin biosynthetic gene cluster

The types of aflatoxin biosynthetic gene clusters in *A. oryzae*, including those in all of our samples (confirmed as non-aflatoxigenic) and strains estimated to be *A. oryzae* in this study, were classified into three groups as defined in a previous study.^6^ Moreover, we found that Kusumoto Group 3 was nested in Group 2, while Group 1 was located far from the other two (Supplementary Fig. S5). Aflatoxin cluster sequences were not clustered by their toxigenicity, suggesting that detoxification in *A. oryzae*/*A. flavus* had occurred in parallel. This is consistent with a previous report inferring that the selective pressure against toxins was lost in nature.^19^ Because the non-toxicity of *A. oryzae* is phylogenetically guaranteed, *tane-koji* manufacturers might have distinguished *A. oryzae* from *A. flavus* based on their growth ability on rice and incidentally selected aflatoxicity.

### 3.6. Concluding remarks

We acquired 82 industrial strains from five Japanese *tane-koji* manufacturers and conducted whole-genome sequencing to determine their draft genomes. Through phylogenetic tree-based inferences of the chromosomal genome and chromosome recombination analysis, we showed that *A. oryzae* strains have undergone multiple inter-genomic recombination events between *A. oryzae* ancestors. However, sexual recombination among domesticated species did not appear to have occurred during the domestication process, at least in the past few decades; therefore, we hypothesized that evolutionary pressure introduced by the domestication of *A. oryzae* is extremely limited to intra-genomic mutation and rearrangements. Through intra- and inter-cladal comparative analysis, we showed that the evolutionary pressure of domestication selectively caused non-synonymous and gap mutations and intra-genomic recombination. Our results suggest that the domestication process might have contributed to altering strain traits while maintaining the activity of industrially useful catalytic enzyme genes by applying selective pressure to peripheral genes involved in fermentation rather than the enzyme-encoding genes themselves.

Our study provides suggestions on the relationship between the evolution and domestication of *A. oryzae*, and importantly, the whole genomic data and phylogenetic tree will help to develop breeding methods based on sexual reproduction using industrial strains.

## Supplementary Material

dsz024_Supplementary_Data

## References

[1] Machida M., Yamada O., Gomi K. 2008, Genomics of *Aspergillus oryzae*: learning from the history of koji mold and exploration of its future, DNA Res., 15, 173–83. 18820080 10.1093/dnares/dsn020 PMC2575883

[2] Machida M., Asai K., Sano M., et al 2005, Genome sequencing and analysis of *Aspergillus oryzae*, Nature, 438, 1157–61. 16372010 10.1038/nature04300

[3] Galagan J.E., Calvo S.E., Cuomo C., et al 2005, Sequencing of *Aspergillus nidulans* and comparative analysis with *A. fumigatus* and *A. oryzae*, Nature, 438, 1105–15. 16372000 10.1038/nature04341

[4] Kobayashi T., Abe K., Asai K., et al 2007, Genomics of *Aspergillus oryzae*, Biosci. Biotechnol. Biochem., 71, 646–70. 17341818 10.1271/bbb.60550

[5] Rokas A., Payne G., Fedorova N.D., et al 2007, What can comparative genomics tell us about species concepts in the genus Aspergillus? Stud. Mycol., 59, 11–7. 18490942 10.3114/sim.2007.59.02 PMC2275189

[6] Kusumoto K.I., Nogata Y., Ohta H. 2000, Directed deletions in the aflatoxin biosynthesis gene homolog cluster of *Aspergillus oryzae*, Curr. Genet., 37, 104–11. 10743566 10.1007/s002940050016

[7] Klich M.A. 2007, *Aspergillus flavus*: the major producer of aflatoxin, Mol. Plant Pathol., 8, 713–22. 20507532 10.1111/j.1364-3703.2007.00436.x

[8] Jørgensen T.R. 2007, Identification and toxigenic potential of the industrially important fungi, *Aspergillus oryzae* and *Aspergillus sojae*, J. Food Prot., 70, 2916–34. 18095455 10.4315/0362-028x-70.12.2916

[9] Geiser D.M., Pitt J.I., Taylor J.W. 1998, Cryptic speciation and recombination in the aflatoxin-producing fungus *Aspergillus flavus*, Proc. Natl. Acad. Sci. U.S.A., 95, 388–93. 9419385 10.1073/pnas.95.1.388 PMC18233

[10] Rokas A. 2009, The effect of domestication on the fungal proteome, Trends Genet., 25, 60–3. 19081651 10.1016/j.tig.2008.11.003

[11] Geiser D.M., Dorner J.W., Horn B.W., Taylor J.W. 2000, The phylogenetics of mycotoxin and sclerotium production in *Aspergillus flavus* and *Aspergillus oryzae*, Fungal Genet. Biol., 31, 169–79. 11273679 10.1006/fgbi.2000.1215

[12] Chang P.-K., Ehrlich K.C., Hua S.-S.T. 2006, Cladal relatedness among *Aspergillus oryzae* isolates and *Aspergillus flavus* S and L morphotype isolates, Int. J. Food Microbiol., 108, 172–7. 16430983 10.1016/j.ijfoodmicro.2005.11.008

[13] Gibbons J.G., Salichos L., Slot J.C., et al 2012, The evolutionary imprint of domestication on genome variation and function of the filamentous fungus *Aspergillus oryzae*, Curr. Biol., 22, 1403–9. 22795693 10.1016/j.cub.2012.05.033 PMC3416971

[14] Geiser D.M., Timberlake W.E., Arnold M.L. 1996, Loss of meiosis in Aspergillus, Mol. Biol. Evol., 13, 809–17. 8754217 10.1093/oxfordjournals.molbev.a025641

[15] Horn B.W., Moore G.G., Carbone I. 2009, Sexual reproduction in *Aspergillus flavus*, Mycologia, 101, 423–9. 19537215 10.3852/09-011

[16] Horn B.W., Gell R.M., Singh R., Sorensen R.B., Carbone I. 2016, Sexual reproduction in *Aspergillus flavus* sclerotia: acquisition of novel alleles from soil populations and uniparental mitochondrial inheritance, PLoS One , 11, e0146169. 26731416 10.1371/journal.pone.0146169 PMC4701395

[17] Wada R., Maruyama J.-I., Yamaguchi H., et al 2012, Presence and functionality of mating type genes in the supposedly asexual filamentous fungus *Aspergillus oryzae*, Appl. Environ. Microbiol., 78, 2819–29. 22327593 10.1128/AEM.07034-11 PMC3318824

[18] Ramirez-Prado J.H., Moore G.G., Horn B.W., Carbone I. 2008, Characterization and population analysis of the mating-type genes in *Aspergillus flavus* and *Aspergillus parasiticus*, Fungal Genet. Biol., 45, 1292–9. 18652906 10.1016/j.fgb.2008.06.007

[19] Chang P.-K., Ehrlich K.C. 2010, What does genetic diversity of *Aspergillus flavus* tell us about *Aspergillus oryzae*? Int. J. Food Microbiol., 138, 189–99. 20163884 10.1016/j.ijfoodmicro.2010.01.033

[20] van Burik J.-A.H., Schreckhise R.W., White T.C., Bowden R.A., Myerson D. 1998, Comparison of six extraction techniques for isolation of DNA from filamentous fungi, Med. Mycol., 36, 299–303. 10075499

[21] Kajitani R., Toshimoto K., Noguchi H., et al 2014, Efficient de novo assembly of highly heterozygous genomes from whole-genome shotgun short reads, Genome Res., 24, 1384–95. 24755901 10.1101/gr.170720.113 PMC4120091

[22] Langmead B., Salzberg S.L. 2012, Fast gapped-read alignment with Bowtie 2, Nat. Methods, 9, 357–9. 22388286 10.1038/nmeth.1923 PMC3322381

[23] Lukashin A., Borodovsky M. 1998, GeneMark.hmm: new solutions for gene finding, Nucleic Acids Res., 26, 1107–15. 9461475 10.1093/nar/26.4.1107 PMC147337

[24] Stanke M., Schöffmann O., Morgenstern B., Waack S. 2006, Gene prediction in eukaryotes with a generalized hidden Markov model that uses hints from external sources, BMC Bioinformatics, 7, 62. 16469098 10.1186/1471-2105-7-62 PMC1409804

[25] Wu T.D., Watanabe C.K. 2005, GMAP: a genomic mapping and alignment program for mRNA and EST sequences, Bioinformatics, 21, 1859–75. 15728110 10.1093/bioinformatics/bti310

[26] Haas B.J., Salzberg S.L., Zhu W., et al 2008, Automated eukaryotic gene structure annotation using EVidenceModeler and the program to assemble spliced alignments, Genome Biol., 9, R7. 18190707 10.1186/gb-2008-9-1-r7 PMC2395244

[27] Simão F.A., Waterhouse R.M., Ioannidis P., Kriventseva E.V., Zdobnov E.M. 2015, BUSCO: assessing genome assembly and annotation completeness with single-copy orthologs, Bioinformatics, 31, 3210–2. 26059717 10.1093/bioinformatics/btv351

[28] Emms D.M., Kelly S. 2015, OrthoFinder: solving fundamental biases in whole genome comparisons dramatically improves orthogroup inference accuracy, Genome Biol., 16, 157. 26243257 10.1186/s13059-015-0721-2 PMC4531804

[29] Kanehisa M., Sato Y., Morishima K. 2016, BlastKOALA and GhostKOALA: KEGG tools for functional characterization of genome and metagenome sequences, J. Mol. Biol., 428, 726–31. 26585406 10.1016/j.jmb.2015.11.006

[30] Quevillon E., Silventoinen V., Pillai S., et al 2005, InterProScan: protein domains identifier, Nucleic Acids Res., 33, W116–120. 15980438 10.1093/nar/gki442 PMC1160203

[31] Katoh K., Standley D.M. 2013, MAFFT multiple sequence alignment software version 7: improvements in performance and usability, Mol. Biol. Evol., 30, 772–80. 23329690 10.1093/molbev/mst010 PMC3603318

[32] Stamatakis A. 2014, RAxML version 8: a tool for phylogenetic analysis and post-analysis of large phylogenies, Bioinformatics, 30, 1312–3. 24451623 10.1093/bioinformatics/btu033 PMC3998144

[33] Li H., Handsaker B., Wysoker A., et al 2009, The sequence alignment/map format and SAMtools, Bioinformatics, 25, 2078–9. 19505943 10.1093/bioinformatics/btp352 PMC2723002

[34] Sato A., Oshima K., Noguchi H., et al 2011, Draft genome sequencing and comparative analysis of *Aspergillus sojae* NBRC4239, DNA Res., 18, 165–76. 21659486 10.1093/dnares/dsr009 PMC3111232

[35] Yin G., Hua S.S.T., Pennerman K.K., et al 2018, Genome sequence and comparative analyses of atoxigenic *Aspergillus flavus* WRRL 1519, Mycologia, 110, 482–93. 29969379 10.1080/00275514.2018.1468201

[36] Toyotome T., Hamada S., Yamaguchi S., et al 2019, Comparative genome analysis of *Aspergillus flavus* clinically isolated in Japan, DNA Res., 26, 95–103. 30520983 10.1093/dnares/dsy041 PMC6379028

[37] Thammarongtham C., Nookaew I., Vorapreeda T., et al 2018, Genome characterization of oleaginous *Aspergillus oryzae* BCC7051: a potential fungal-based platform for lipid production, Curr. Microbiol., 75, 57–70. 28865010 10.1007/s00284-017-1350-7

[38] Zhao G., Yao Y., Hou L., Wang C., Cao X. 2014, Draft genome sequence of *Aspergillus oryzae* 100-8, an increased acid protease production strain, Genome Announc., 2, pii: e00548–14. 24903875 10.1128/genomeA.00548-14 PMC4047454

[39] Bayman P., Cotty P.J. 1991, Vegetative compatibility and genetic diversity in the *Aspergillus flavus* population of a single field, Can. J. Bot., 69, 1707–11.

[40] Umemura M., Koike H., Yamane N., et al 2012, Comparative genome analysis between *Aspergillus oryzae* strains reveals close relationship between sites of mutation localization and regions of highly divergent genes among Aspergillus species, DNA Res., 19, 375–82. 22912434 10.1093/dnares/dss019 PMC3473370

[41] Mayorga M.E., Timberlake W.E. 1992, The developmentally regulated *Aspergillus nidulans* wA gene encodes a polypeptide homologous to polyketide and fatty acid synthases, Molec. Gen. Genet., 235, 205–12. 1465094 10.1007/BF00279362

[42] Fernandez E.Q., Moyer D.L., Maiyuran S., Labaro A., Brody H. 2012, Vector-initiated transitive RNA interference in the filamentous fungus *Aspergillus oryzae*, Fungal Genet. Biol., 49, 294–301. 22366516 10.1016/j.fgb.2012.01.011

[43] Bok J.W., Keller N.P. 2004, LaeA, a regulator of secondary metabolism in Aspergillus spp, Eukaryot. Cell, 3, 527–35. 15075281 10.1128/EC.3.2.527-535.2004 PMC387652

[44] Oda K., Kobayashi A., Ohashi S., Sano M. 2011, *Aspergillus oryzae* laeA regulates kojic acid synthesis genes, Biosci. Biotechnol. Biochem., 75, 1832–4. 21897021 10.1271/bbb.110235

[45] Herrera M.L., Vallor A.C., Gelfond J.A., Patterson T.F., Wickes B.L. 2009, Strain-dependent variation in 18S ribosomal DNA copy numbers in *Aspergillus fumigatus*, J. Clin. Microbiol., 47, 1325–32. 19261786 10.1128/JCM.02073-08 PMC2681831

[46] Yoshino-Yasuda S., Fujino E., Matsui J., Kato M., Kitamoto N. 2013, Molecular analysis of the α-amylase gene, AstaaG1, from shoyu koji mold, *Aspergillus sojae* KBN1340, Food Sci. Technol. Res., 19, 255–61.

