# Supplementary material for: Evolution of Aspergillus oryzae before and after domestication inferred by large-scale comparative genomic analysis
Source: DNA Res. 2019 Nov 22;26(6):465–72. doi: 10.1093/dnares/dsz024 (PMC6993814; doi:10.1093/dnares/dsz024)
Supplement: dsz024_Supplementary_Data [file dsz024_supplementary_data.zip › dsz024-Suppl_data/Supplementary_Data.pdf]

Full text of methods

2.1 Sample collection and DNA preparation

For genomic sequencing, 82 *A. oryzae* and three *A. sojae* industrially used strains were collected from five independent *tane-koji* (rice malt) manufacturers in different locations in Japan (Table S1). *A. sojae* is an industrial strain closely related to *A. oryzae*, used as an out-group in this study. The *tane-koji* manufacturers have their own isolates and have not shared them for several decades. The strains were inoculated on potato dextrose agar and incubated at 30°C for 2–3 days. Cultured fungal hyphae/spores were suspended in approximately 50 mL of YPD medium and incubated at 30°C for 1–3 days with shaking. From the cultured hyphae, fungal cells were collected, washed with sterilized milliQ water, and stored at -85°C. Whole genomic DNA was extracted using the modified CTAB method based on "Extraction method5".[1] The follow modifications were employed: before CTAB incubation, some of the strains were pre-incubated at 37°C for 60 min with yatalase buffer [0.2% yatalase (Takara Bio, Inc., Shiga, Japan), 10 mM Tris-HCl, 50 mM EDTA, 0.2M NaCl]. RNA was degraded using Ribonuclease (DNase-free) Glycerol Solution (NIPPON GENE, Co., Ltd., Tokyo, Japan) or RNase Cocktail Enzyme Mix (Thermo Fisher Scientific, Waltham, MA, USA) at 37°C for 3 h. A purified genome solution was obtained using Genomic DNA Clean & Concentrator (Zymo Research Corp., Irvine, CA, USA).

2.2 Genome sequencing and assembly

For genome assembly, fragmented genome libraries were prepared from 2.4 μg genomic DNA sheared to 350 base pairs (bp) (for run No. 1) and 550 bp (for run No. 2–5) on average using the TruSeq DNA PCR-Free Library Prep kit (Illumina, Inc., San Diego, CA, USA). The genome libraries were sequences on an Illumina Hiseq2500 using 150 bp (for run No. 1) and 250 bp (for run No. 2–5) paired-end runs at the National Institute of Genetics (Shizuoka, Japan).

Low-quality regions and adaptor sequences were filtered out from the paired-end reads with Platanus_trim v1.0.7 and high-quality reads were assembled with Platanus v1.2.4 using default parameters.[2] Only paired-end reads were used for contig assembly, scaffolding, and gap-closure. To remove contamination, genomic scaffolds ≥1,000 bp were aligned to RIB40 chromosomal and mitochondrial sequences downloaded from NCBI GenBank (GCA_000184455.3) with LAST v869[3] and to RefSeq DB release 89[4] with BLASTn v2.2.8.[5] The scaffolds aligned to bacterial genomes or the mitochondrial genome of RIB40 were removed.

2.3 Gene prediction and orthologous clustering

For comparative genomic analysis, 152 genomic scaffolds (85 from our samples and 67 from NCBI GenBank, containing contigs not scaffolded) of the newly sequenced or NCBI GenBank *A. oryzae*, *A. flavus*, *A. sojae*, and *A. parasiticus* strains were used (Table S2). "*A. oryzae* ATCC12892" (GCA_002894705.1)[6] was confirmed as *A. sojae* by phylogenetic analysis, and thus was re-annotated to *A. sojae* in this study.

Gene coding regions were predicted using two methods: GeneMark-ES v4.32[7] (gmes_petap.pl --ES --fungus) and AUGUSTUS v3.3[8] (--species=*Aspergillus_oryzae*) for ab initio prediction and GMAP v2017-11-1524[9] for reference-based prediction (references, *A. oryzae* RIB40 s01-m08-r29, *A. niger* CBS_513.88 s01-m07-r04, and *A. flavus* NRRL_3357 were downloaded from the *Aspergillus* Genome Database (AspGD),[10] http://www.aspgd.org). The results of gene prediction were fixed into GFF files and combined with EVidenceModeler v1.1.1[11] using following weight parameters, "*A. oryzae* RIB40 s01-m08-r29": 10, "*A. niger* CBS 513.88 s01-m07-r04" / "*A. flavus* NRRL_3357": 5, "AUGUSTUS": 2, and "GeneMark-ES": 1. From the combined GFF files, FASTA files of the protein sequences of the genes were obtained with gffread v0.9.10,[12] removing genes that were frame-shifted or lacking start/stop codons. To

confirm the accuracy of prediction of open reading frame regions and annotate chromosomal loci, *A. oryzae* RIB40 s01-m08-r29 and s01-m09-r06 protein sequences downloaded from AspGD were added. The predicted protein sets of each strain and reference protein sets (*A. oryzae* RIB40 s01-m08-r29 and s01-m09-r06) were evaluated with BUSCO v3.0.128 using 4,046 core genes from eurotiomycetes_odb9.[13]

Ortholog clustering (Markov clustering) for all protein sequences of *A. oryzae*, *A. flavus*, *A. sojae*, and *A. parasiticus* strains were performed with OrthoFinder v2.2.6[14] using diamond v0.9.22[15] as the alignment software. All proteins were tagged with an orthogroup ID (OG). Representative protein sequences (the revised protein sequence, as described in Section 2.4.5) for each OG were annotated with GhostKOALA v2.2[16] for protein function and InterProScan v5.28-67.0[17] for protein motif/domain searching.

2.4 Comparative genomics

2.4.1 Multiple alignment and phylogenetic inference with single copy OGs

From the 152 gene sets for which ortholog clustering was performed, the common single copy OGs (common single-copy genes (SCGs)) were determined. For gene sequence comparison, exon-intron-concatenated sequences were extracted to eliminate the influence of misprediction of exon/intron. Degapped gene sequences (DGSs) were generated from multiple alignments for each common SCG generated with MAFFT v7.2.2.2;[18] columns containing at least one gap or one ambiguous base ("N" or degenerate bases) were removed. The DGS of each common SCG tandemly concatenated. Maximum-likelihood based phylogenetic inference of each concatenated DGS was performed with RAxML v8.2.11[19] using the options -m GTRGAMMAI -N 100. The phylogenetic tree file (Newick) was visualized with FigTree v1.4.3 and manually rerooted. In subsequent analysis, the strains were grouped into the clades defined in this study.

2.4.2 Phylogenetic inference of aflatoxin cluster OGs

The aflatoxin synthetic cluster region was determined by local alignment of the RIB40 genome (GCA_000184455.3) and aflatoxin cluster sequence of *A. flavus* AF36 (AY510455.1) using BLASTn v2.2.8. For comparative analysis, a concatenated DGS tree of 19 SCGs in the aflatoxin synthetic cluster (AO090026000008–AO090026000036) was generated. Missing genes were altered by full gaps not to contribute to phylogenetic inference. Phylogenetic inference was performed with RAxML v8.2.11 using the options -m GTRGAMMAI -N 100.

2.4.3 Visualization of putative genetic recombination

To visualize intercladal recombination, assuming that exactly the same sequences were derived from the same ancestor, strain-clade similarities along the genome positions were calculated. First, all DGSs were dereplicated, clustered by exact matching, within each SCG (common SCG and allowing gene deletion) as described in section 2.4.1. Each SCG was assigned information for the genome position in the RIB40 genome. Second, for single nucleotide-level comparison, similarities for each SCG were calculated using a binary distance; if strain "X" shared the identical sequence as at least one strain in clade "Y", which differed from the clade to which strain "X" belonged, the similarity score between "X" and "Y" was set to 1 at that gene position; otherwise, it was set to 0. Finally, to smooth the results, the average scores were calculated within the windows along the genome position (window_size=100,000 bp, interval_size=50,000 bp), only when the window contained at least two genes. For each window, the closest clade was identified and visualized as a color-code. Only SCGs present in RIB40 (12,203 genes, predicted on the eight chromosomes) were used for visualization.

2.4.4 Determination of mating-type

The reference primer sequences for MAT-type[20] were mapped to the genome sequences with bowtie2[21] using the parameters --sensitive-local --no-discordant --no-mixed -X 2000 --end-to-end.

2.4.5 Analysis of intracladal mutation pressure

To reduce false detection, 101 strains with BUSCO scores of more than 96% were used in this analysis.

To estimate inter- and intracladal mutation pressure, revised coding sequences (RCSs) were generated; if a sequence lacked either or both ends in the alignment, the gene region was reacquired, with the length adjusted to the longest sequence in the alignment. The genes whose sequences could not be extended due to the location at the edge of the contig were treated as deleted. Then, to remove fluctuation of exon prediction, RCSs were determined the consensus coding sequences, most frequently predicted exon regions, while revised protein sequences (RPSs) were translated from the RCSs. The consensus coding sequence was determined with non-gapped sequences within each group, and the sequences with Hamming distance > 1% were regarded as a paralog and removed. Finally, the numbers of synonymous/non-synonymous mutations and gaps in the RCSs were calculated within species/clades. Consecutive gaps were counted as one gap.

The number of intracladal synonymous/non-synonymous/gap mutations were calculated as total mutations of clade A–H, and then the number of intercladal mutations were estimated as the difference between the number of intraspecific mutations (within *A. oryzae*) and intracladal mutations. If the number of intraspecific mutations were less than that of intracladal mutations due to mis-clustering of homologs, especially with unremoved closely-related paralogs

(Hamming distance < 1%), the calculated number of intercladal mutations would be < 0, so those were not used for the following statistical tests. The event that the same mutation occurred between different clades was not considered because it was hardly distinguished from mis-clustering.

To test the neutrality of the mutation, Fisher's exact test was performed for the number of mutations within OG and its total. Synonymous mutations vs. non-synonymous mutation or gap mutations vs. synonymous mutations, between intercladal and intracladal mutations were tested.

Synteny block (SB) and non-synteny block (NSB) were annotated to each gene using ortholog assignment result downloaded from AspGD[10] (A_oryzae_RIB40_A_fumigatus_Af293_orthologs.txt, A_oryzae_RIB40_A_nidulans_FGSC_A4_orthologs.txt). When a gene had an orthologous gene with at least one of *A. fumigatus* and *A. nidulans*, it was regarded as a SB gene, otherwise a NSB gene.

2.4.6 Sequence depth analysis for detecting intracladal gene duplication/deletion

Chromosomal duplication and deletion were inferred by direct read-mapping to the RIB40 genome. Only forward reads mapped using bowtie2 v2.2.6 with --very-sensitive and --end-to-end mode, depths were calculated with samtools v1.3.1[22] using the "samtools depth" command with default parameters. The sequence depths at each genomic position were normalized by the median of non-zero values. From normalized depth, copy number of genes were estimated. To identify gene regions, "A_*oryzae*_RIB40_version_s01-m09-r06_chromosomal_feature.tab" downloaded from AspGD was used. The mapping depths of non-single-copy genes in the RIB40 genome were grouped by OGs.

1. van Burik, J.-A. H., Schreckhise, R. W., White, T. C., Bowden, R. A., and Myerson, D. 1998, Comparison of six extraction techniques for isolation of DNA from filamentous fungi.

Med. Mycol., **36**, 299–303.

2. Kajitani, R., Toshimoto, K., Noguchi, H., et al. 2014, Efficient de novo assembly of highly heterozygous genomes from whole-genome shotgun short reads. *Genome Res.*, **24**, 1384–1395.

3. Kiełbasa, S. M., Wan, R., Sato, K., Horton, P., and Frith, M. C. 2011, Adaptive seeds tame genomic sequence comparison. *Genome Res.*, **21**, 487–493.

4. Pruitt, K. D., Tatusova, T., and Maglott, D. R. 2007, NCBI reference sequences (RefSeq): a curated non-redundant sequence database of genomes, transcripts and proteins. *Nucleic Acids Res.*, **35**, D61–D65.

5. Altschul, S., Madden, T. L., Schäffer, A. A., et al. 1997, Gapped BLAST and PSI-BLAST: a new generation of protein database search programs. *Nucleic Acids Res.*, **25**, 3389–3402.

6. Deng, S., Pomraning, K. R., Bohutskyi, P., and Magnuson, J. K. 2018, Draft Genome Sequence of Aspergillus oryzae ATCC 12892. *Genome Announc.*, **6**, e00251-18.

7. Lukashin, A., and Borodovsky, M. 1998, GeneMark.hmm: new solutions for gene finding. *Nucleic Acids Res.*, **26**, 1107–1115.

8. Stanke, M., Schöffmann, O., Morgenstern, B., and Waack, S. 2006, Gene prediction in eukaryotes with a generalized hidden Markov model that uses hints from external sources. *BMC Bioinformatics*, **7**, 62.

9. Wu, T. D., and Watanabe, C. K. 2005, GMAP: a genomic mapping and alignment program for mRNA and EST sequences. *Bioinformatics*, **21**, 1859–1875.

10. Arnaud, M. B., Cerqueira, G. C., Inglis, D. O., et al. 2012, The *Aspergillus* Genome Database (AspGD): recent developments in comprehensive multispecies curation, comparative genomics and community resources. *Nucleic Acids Res.*, **40**, D653–D659.

11. Haas, B. J., Salzberg, S. L., Zhu, W., et al. 2008, Automated eukaryotic gene structure annotation using EVidenceModeler and the Program to Assemble Spliced Alignments. *Genome Biol.*, **9**, R7.

12. Trapnell, C., Roberts, A., Goff, L., et al. 2012, Differential gene and transcript expression analysis of RNA-seq experiments with TopHat and Cufflinks. *Nat. Protoc.*, **7**, 562–78.

13. Simão, F. A., Waterhouse, R. M., Ioannidis, P., Kriventseva, E. V., and Zdobnov, E. M. 2015, BUSCO: assessing genome assembly and annotation completeness with single-copy orthologs. *Bioinformatics*, **31**, 3210–3212.

14. Emms, D. M., and Kelly, S. 2015, OrthoFinder: solving fundamental biases in whole genome comparisons dramatically improves orthogroup inference accuracy. *Genome Biol.*, **16**, 157.

15. Buchfink, B., Xie, C., and Huson, D. H. 2015, Fast and sensitive protein alignment using DIAMOND. *Nat. Methods*, **12**, 59–60.

16. Kanehisa, M., Sato, Y., and Morishima, K. 2016, BlastKOALA and GhostKOALA: KEGG Tools for Functional Characterization of Genome and Metagenome Sequences. *J. Mol. Biol.*, **428**, 726–731.

17. Quevillon, E., Silventoinen, V., Pillai, S., et al. 2005, InterProScan: protein domains identifier. *Nucleic Acids Res.*, **33**, W116–W120.

18. Katoh, K., and Standley, D. M. 2013, MAFFT Multiple Sequence Alignment Software Version 7: Improvements in Performance and Usability. *Mol. Biol. Evol.*, **30**, 772–80.

19. Stamatakis, A. 2014, RAxML version 8: a tool for phylogenetic analysis and post-analysis of large phylogenies. *Bioinformatics*, **30**, 1312–1313.

20. Wada, R., Maruyama, J.-I., Yamaguchi, H., et al. 2012, Presence and functionality of mating type genes in the supposedly asexual filamentous fungus *Aspergillus oryzae*. *Appl.*

Environ. Microbiol., **78**, 2819–2829.

21. Langmead, B., and Salzberg, S. L. 2012, Fast gapped-read alignment with Bowtie 2. *Nat. Methods*, **9**, 357–359.

22. Li, H., Handsaker, B., Wysoker, A., et al. 2009, The Sequence Alignment/Map format and SAMtools. *Bioinformatics*, **25**, 2078–2079.
