# Supplementary material for: Evolution of Aspergillus oryzae before and after domestication inferred by large-scale comparative genomic analysis
Source: DNA Res. 2019 Nov 22;26(6):465–72. doi: 10.1093/dnares/dsz024 (PMC6993814; doi:10.1093/dnares/dsz024)
Supplement: dsz024_Supplementary_Data [file dsz024_supplementary_data.zip › dsz024-Suppl_data/Supplementary_Figure.pdf]

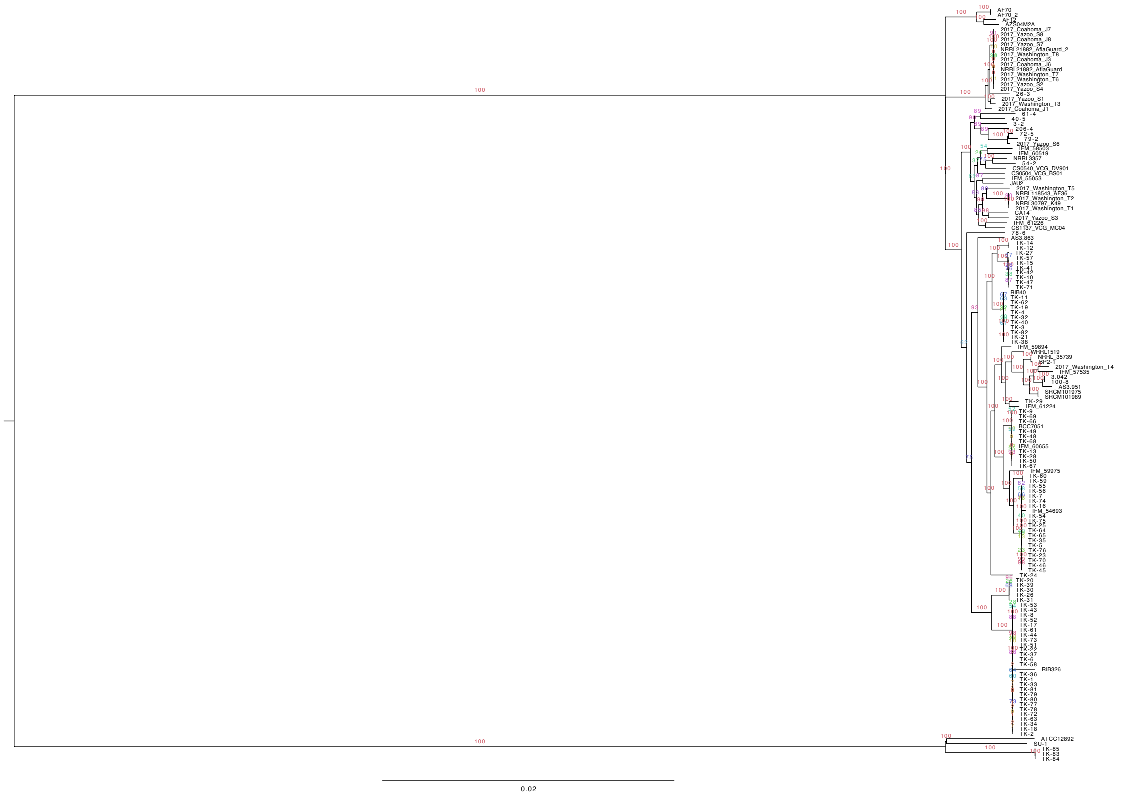

Fig. S1 Full figure of phylogenetic tree of concatenated degapped gene sequences (DGSs).

147 strains of *Aspergillus oryzae*, *Aspergillus flavus* and five out-group strains of *Aspergillus sojae* and *Aspergillus parasitucus.*

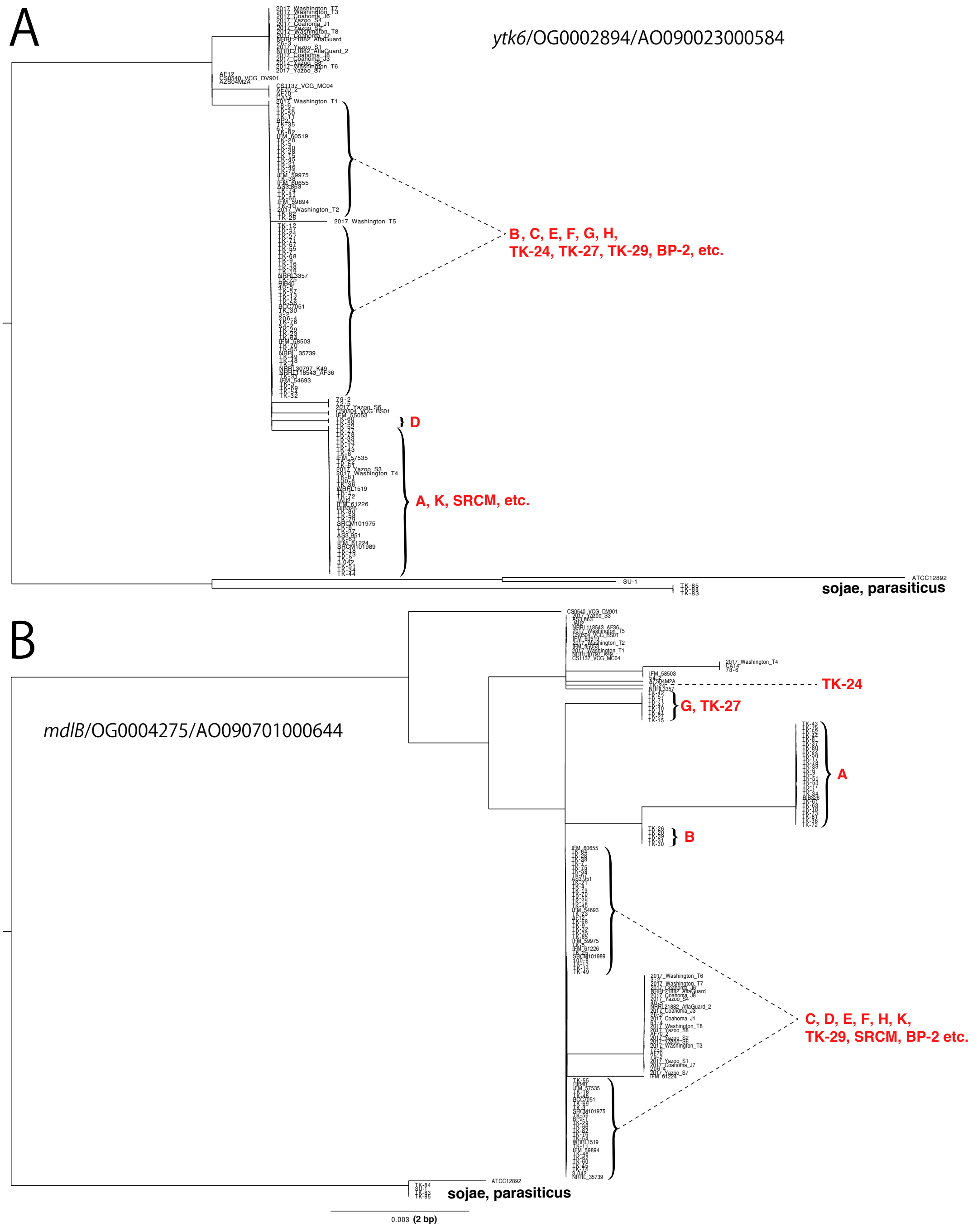

Fig. S2-1 Phylogenetic tree of two genes, *ytk6* and *mdlB*.

Curly brackets show the exact same sequences with clade/strain names of *Aspergillus oryzae*.

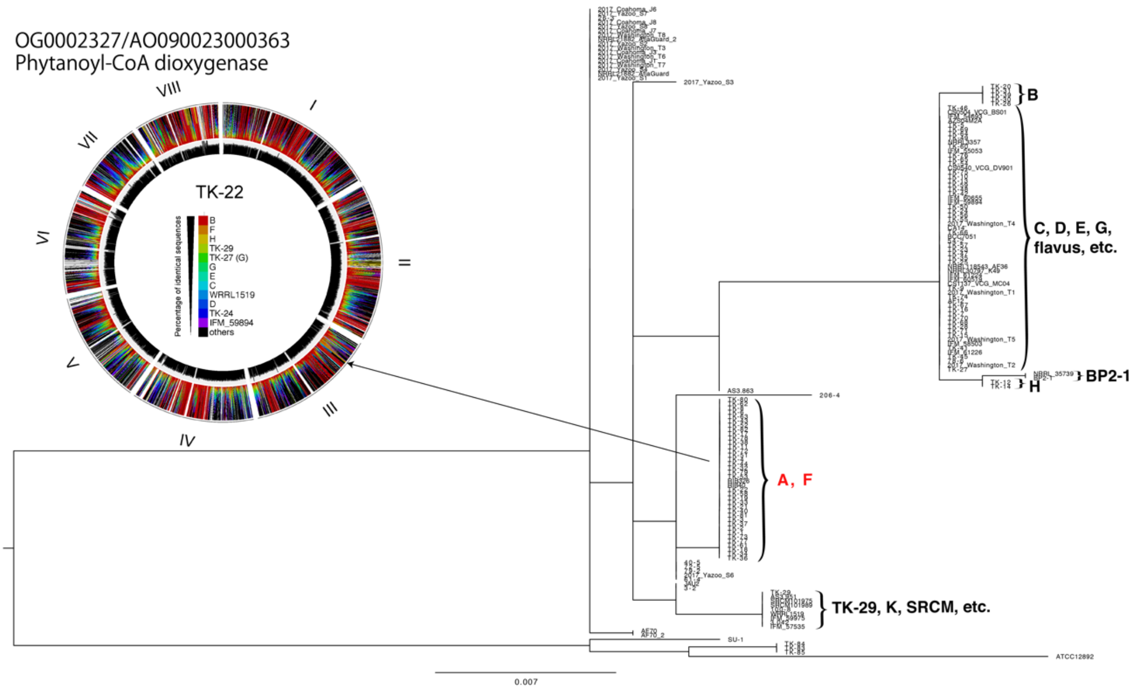

Fig. S2-2 Phylogenetic tree of OG0002327, a gene with identical clade A and F sequences

In the genome mixture of TK-22 (Fig S3-1), the region on which OG0002327 was located was exclusively homologous to clade F.

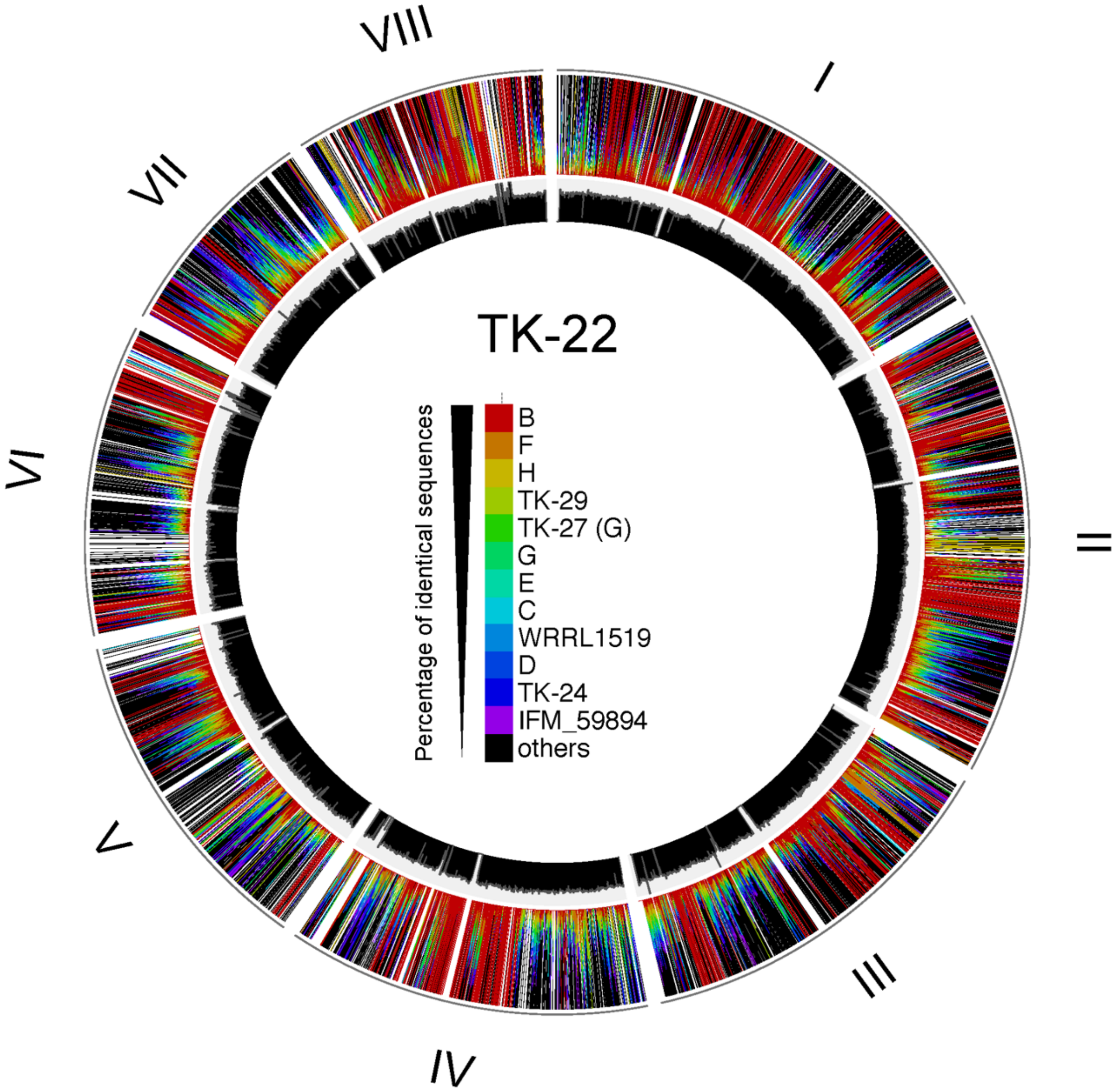

Fig S3-1 Unsmoothed figure of the chromosomal mixture of TK-22 (clade A).

Each bar shows the clades having the exact same sequence for each gene. White bars show gene deletions or genes unique to the clade TK-22 belongs to.

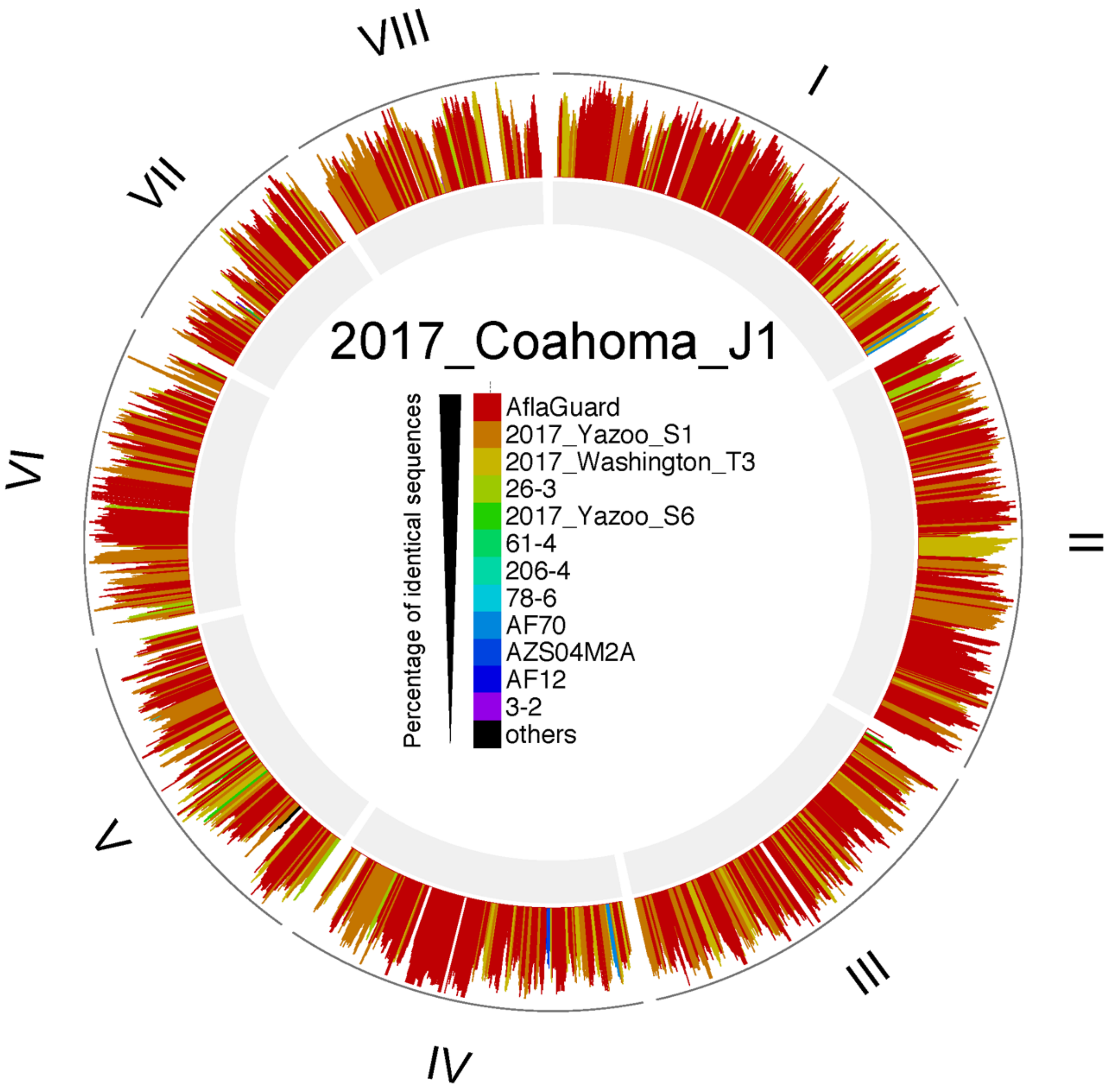

Fig. 3-2 Visualized chromosomal mixture of 2017_Coahoma_J1

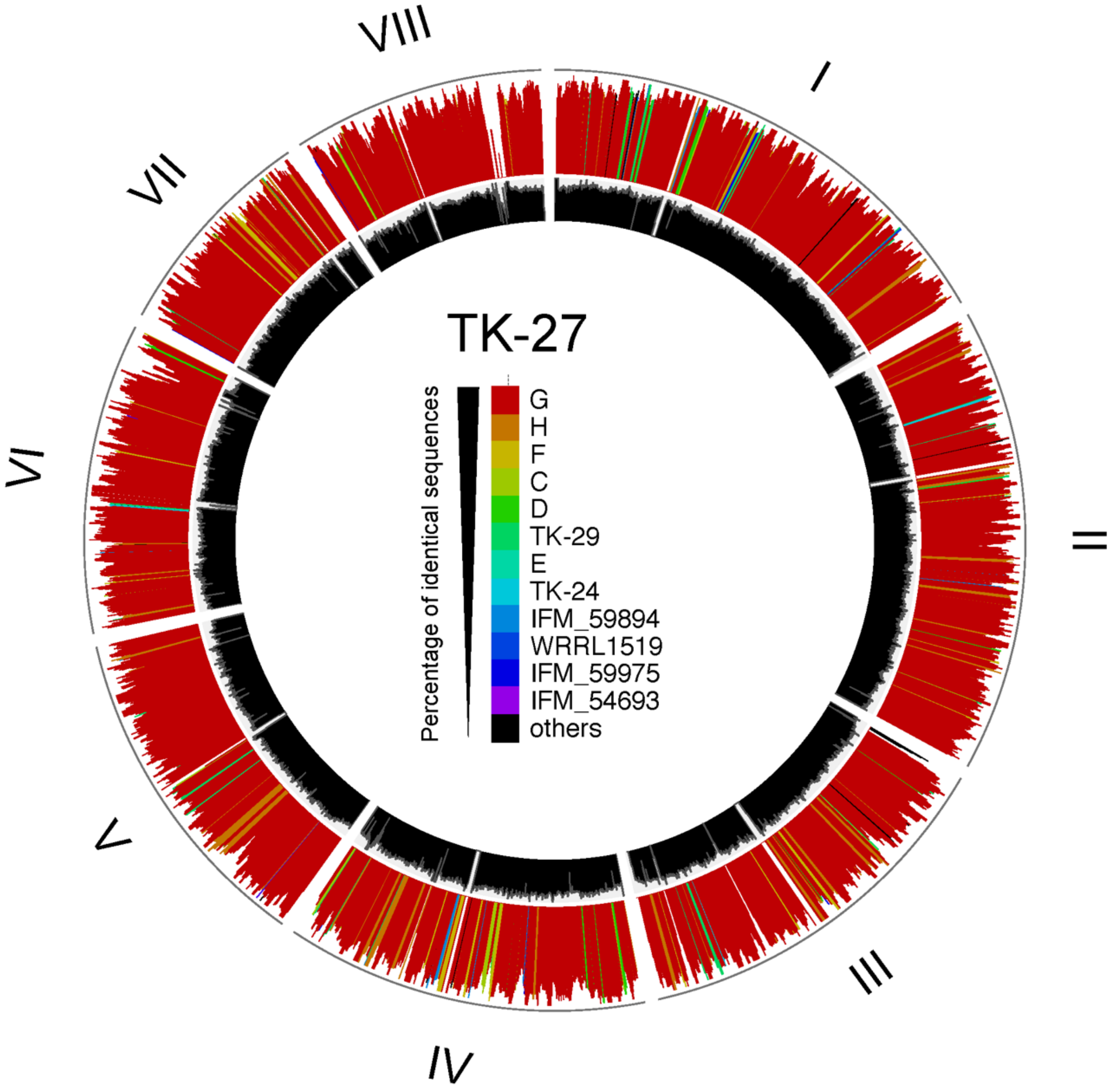

Fig. 3-3 Visualized chromosomal mixture of TK-27

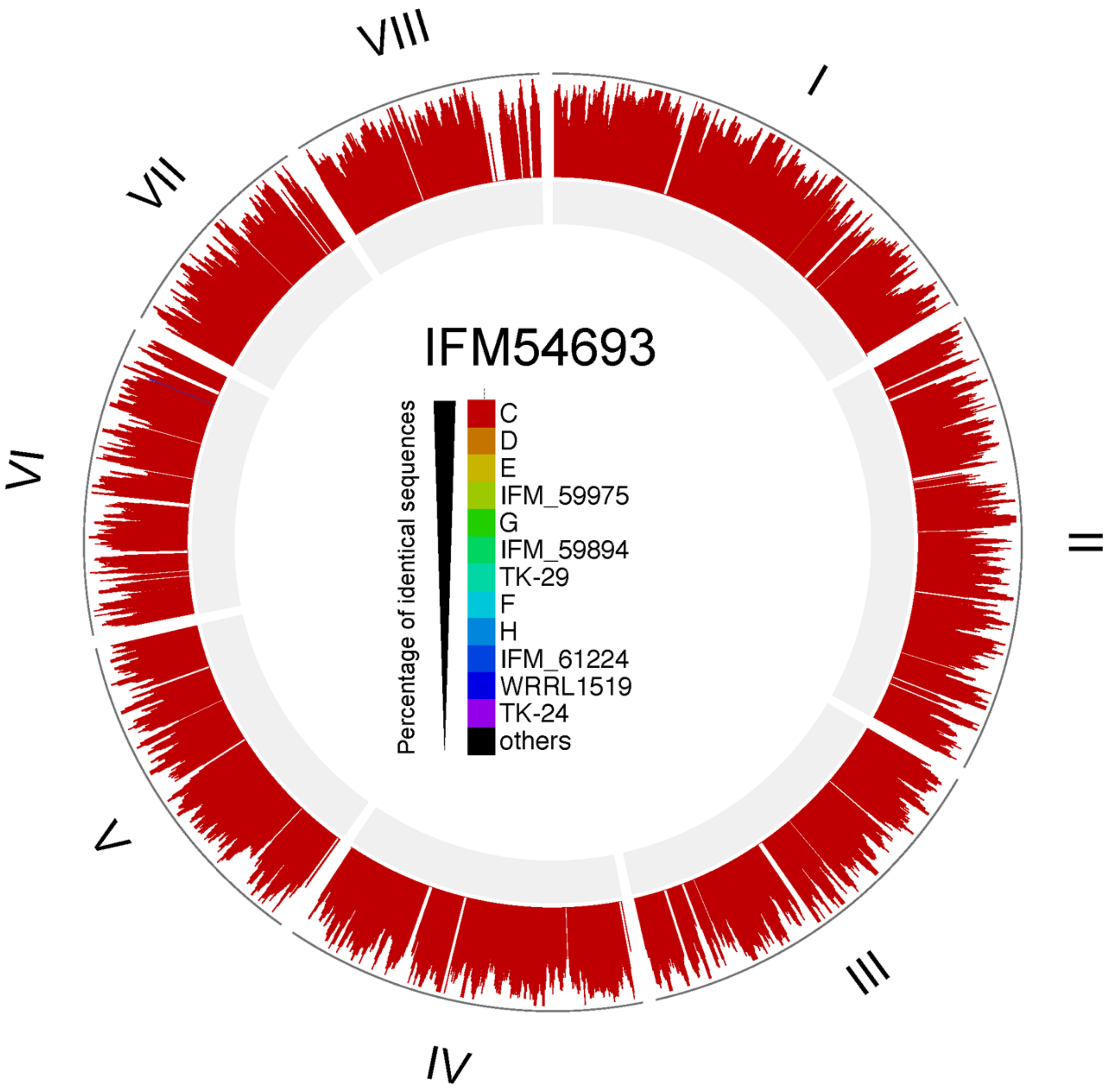

Fig. 3-4 Visualized chromosomal mixture of IFM54693 without excluding clade C, the clade to which the strain belongs.

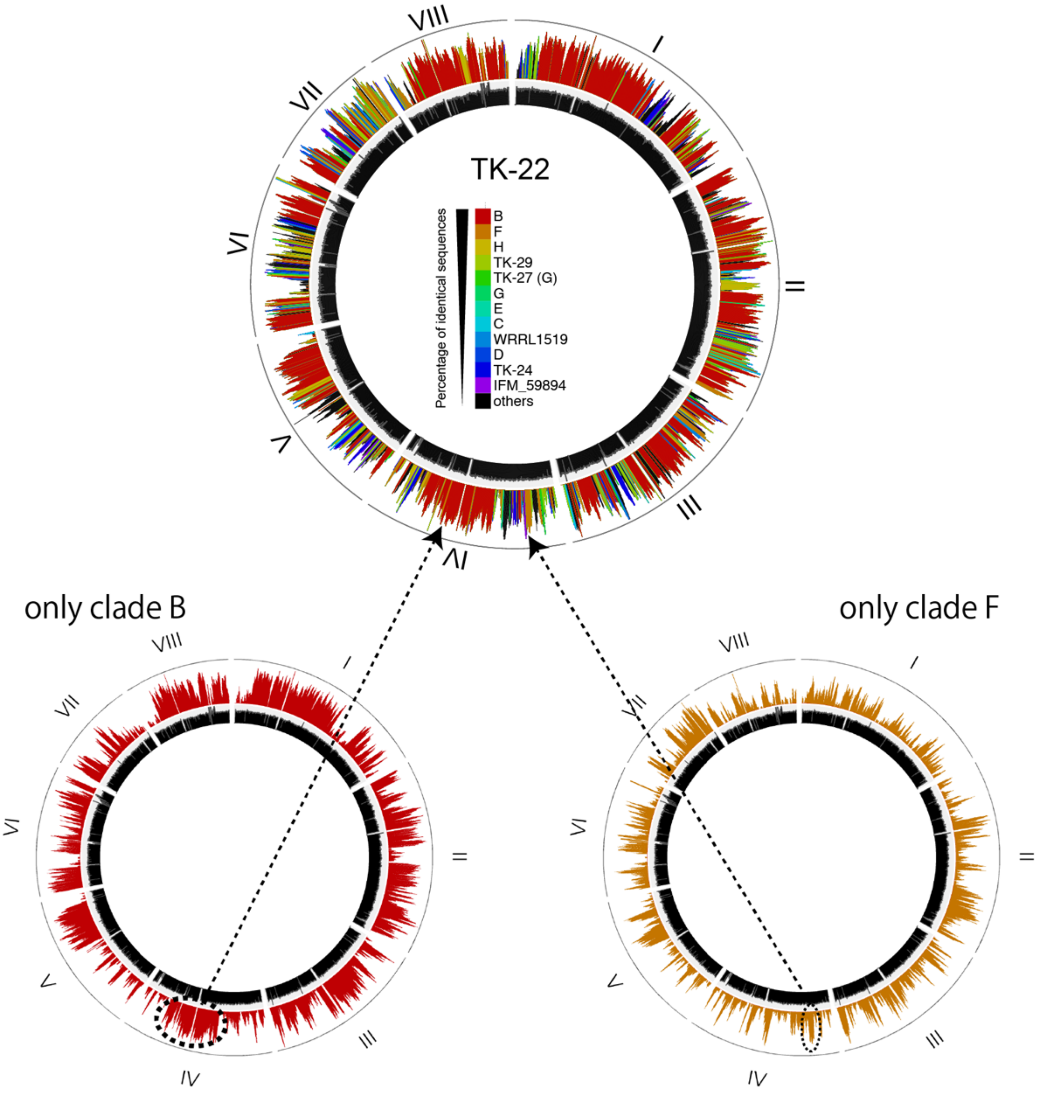

Fig. S3-5 Syntenic regions of TK-22: indivisual comparison with clade B and F.
Dotted circles/lines show examples of selection of synteny regions.

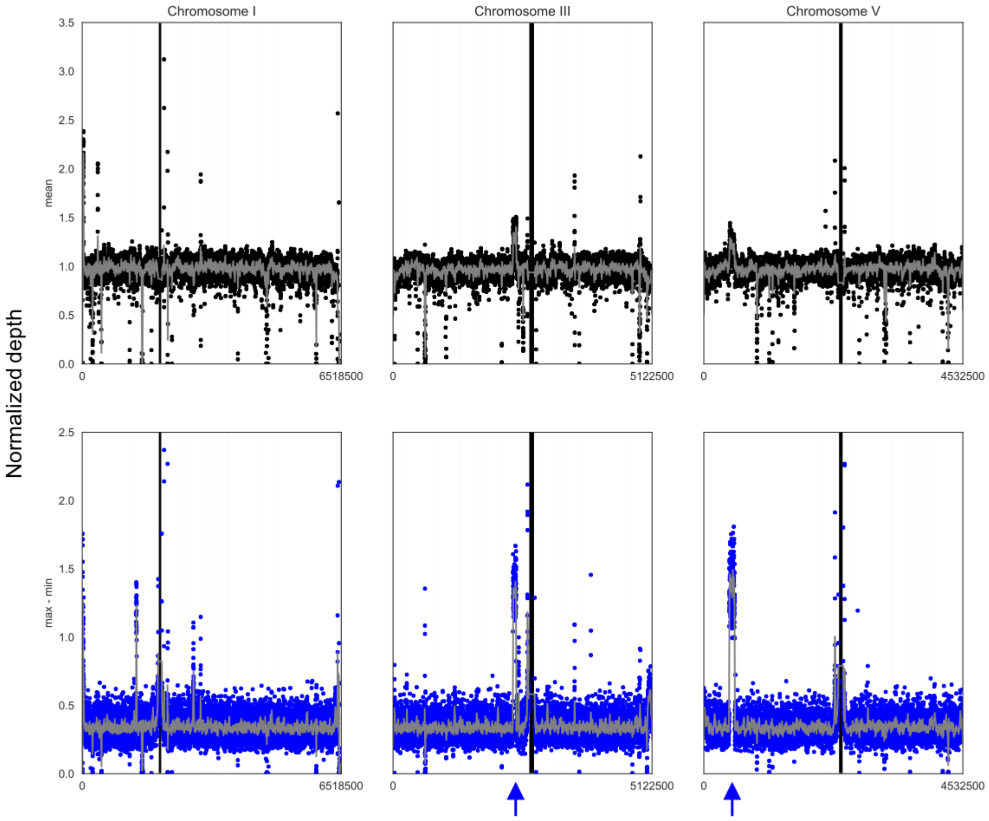

Fig. S4-1 Normalized sequence depth of chromosome I, III, V for the 17 strains of clade C.

Blue arrows indicate large consecutive duplication of about 50,000 bp-regions including tRNA genes. Gray lines show moving average with a 10,000 bp-window. Unassembled region (scaffold gaps) of the reference genome (RIB40) were masked with black boxes.

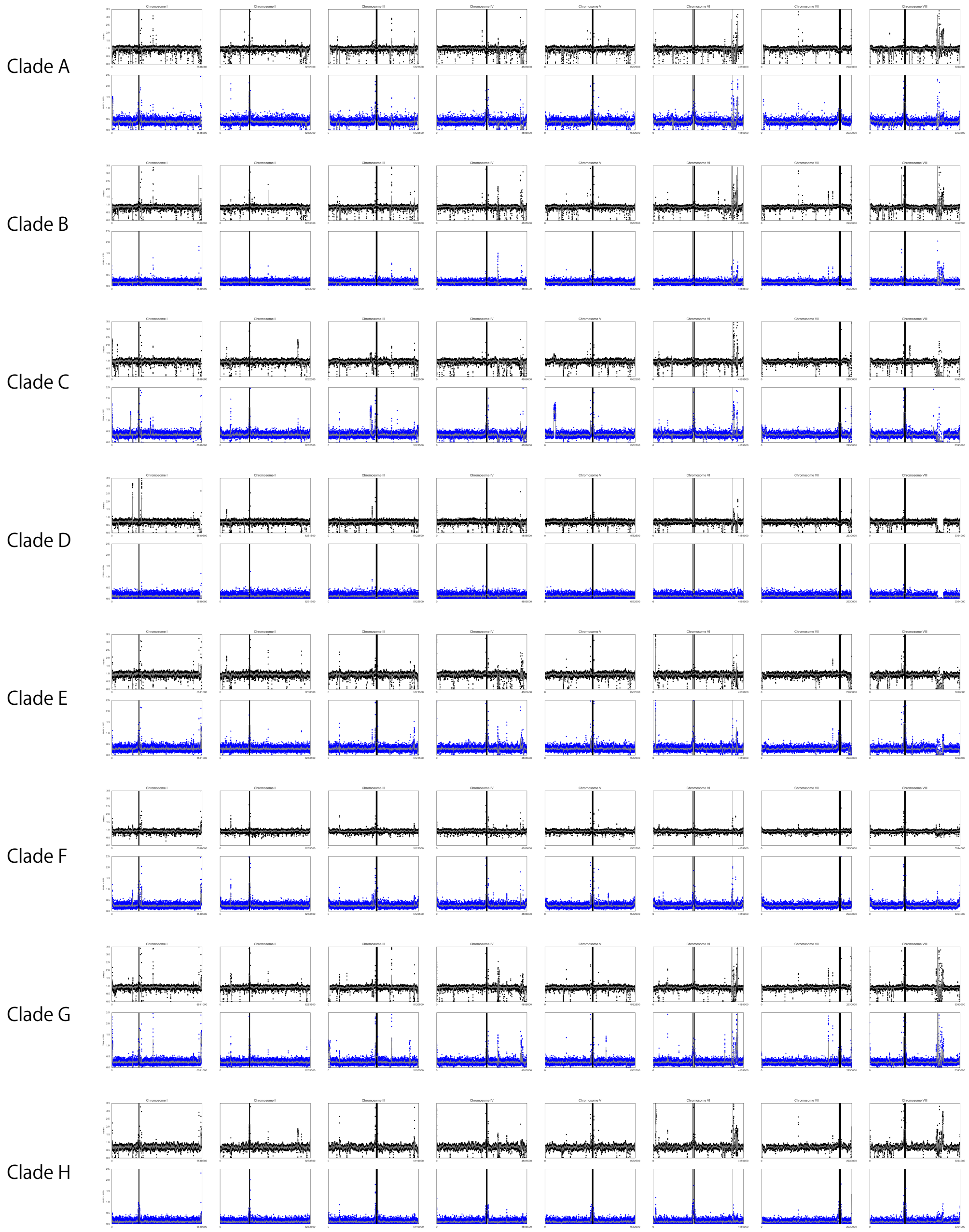

Fig. S4-2 Normalized sequence depth of chromosome I–VIII for *A. oryzae* clade A–H.
Gray lines show moving average with a 10,000 bp-window. Unassembled region (scaffold gaps) of the reference genome (RIB40) were masked with black boxes.

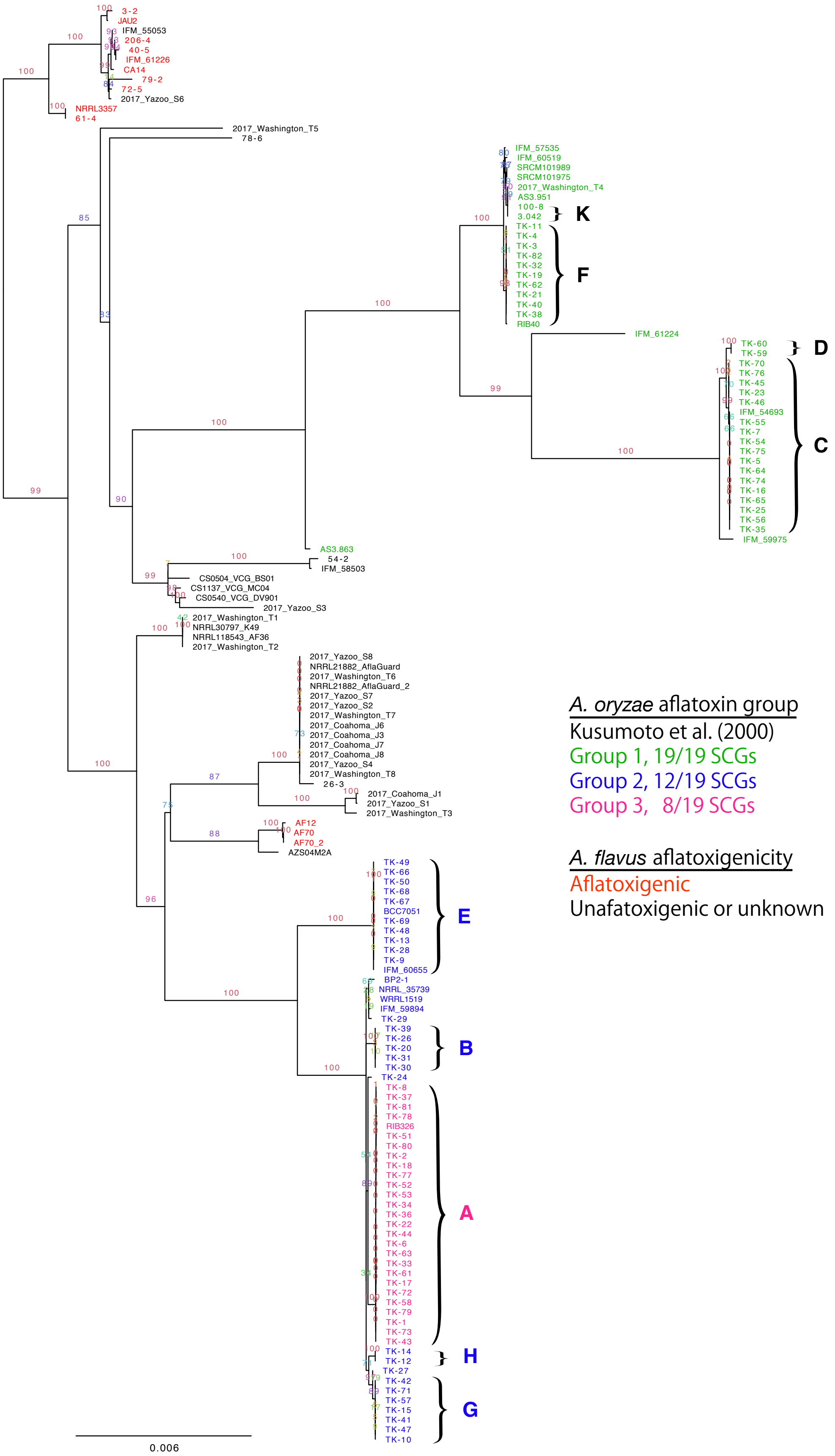

Fig. S5 Phylogenetic tree of 19 genes among the aflatoxin synthetic gene cluster.
